# Baseline Mental Health Status and Early Psychiatric Outcomes after Metabolic-Bariatric Surgery: A Retrospective Cohort Study

**DOI:** 10.1007/s11695-026-08918-5

**Published:** 2026-08-29

**Authors:** Patricia M. Ortega, Luke Boyle, Dana Mahdi, Zahra Al-Essah, Ali Abdul Hussein, Mina Abdul Hussein, Aslesha Deshraju, Elena Brachimi, Prasidha Rai, Elsie Ampomah, Neha Hussain, Faizan Kiani, Mary Obigbesan, Lucy Tweedlie, Samantha Scholtz, Sanjay Purkayastha, Christos Tsironis, Sherif Hakky, Ravi Aggarwal, Ahmed R. Ahmed, Tricia Tan, Julia Kenkre, Kleopatra Alexiadou, Saira Hameed, Harvinder Chahal, Jonathan Cousins, Chioma Izzi-Engbeaya

**Affiliations:** 1 Department of Surgery, Imperial College Healthcare NHS Trust, London, UK; 2 Department of Endocrinology, Barts Health NHS Trust, London, UK; 3 Department of Endocrinology, Imperial College Healthcare NHS Trust, London, UK; 4 Imperial College School of Medicine, Imperial College London, London, UK; 5 Specialist Services, West London Mental Health NHS Trust, London, UK; 6 Department of Life Sciences, Brunel University London, London, UK; 7 Department of Surgery and Cancer, Imperial College London, London, UK; 8 Section of Investigative Medicine and Endocrinology. Department of Metabolism, Digestion and Reproduction, Imperial College London, London, UK

## Abstract

**Objective:**

To evaluate whether documented preoperative mental health (MH) conditions were associated with early postoperative psychiatric events and metabolic outcomes after primary metabolic-bariatric surgery (MBS).

**Methods:**

A retrospective cohort study of adults who underwent primary MBS at a tertiary UK bariatric centre between April 2021 and March 2024 was conducted. Preoperative MH conditions and postoperative outcomes were identified via review of electronic health records. Comparisons between groups were analysed using Fisher’s exact test and regression models adjusted for age, sex, preoperative BMI, and surgery type.

**Results:**

Among 482 patients, 217 (45.0%) had at least one documented preoperative MH condition. During 2-year follow-up, suicidal ideation was recorded in 6/217 (2.8%) in the MH group versus 1/265 (0.4%) in the non-MH group and self-harm (including suicide attempt) in 5/217 (2.3%) versus 1/265 (0.4%), respectively. Preoperative MH conditions were associated with higher odds of postoperative suicidal ideation (OR 6.75, 95% CI 1.09–41.86; *p* = 0.0420), while the association with self-harm was directionally similar but not statistically significant (OR 5.10, 95% CI 0.80–32.72; *p* = 0.0860). Weight-loss and metabolic outcomes did not differ materially by baseline MH status.

**Conclusions:**

In this single-centre cohort, patients with documented preoperative MH conditions achieved comparable metabolic benefit after surgery but had higher recorded early suicidality-related events. Although causality cannot be inferred, these findings support structured postoperative MH follow-up.

**Graphical Abstract:**

**Figure:**
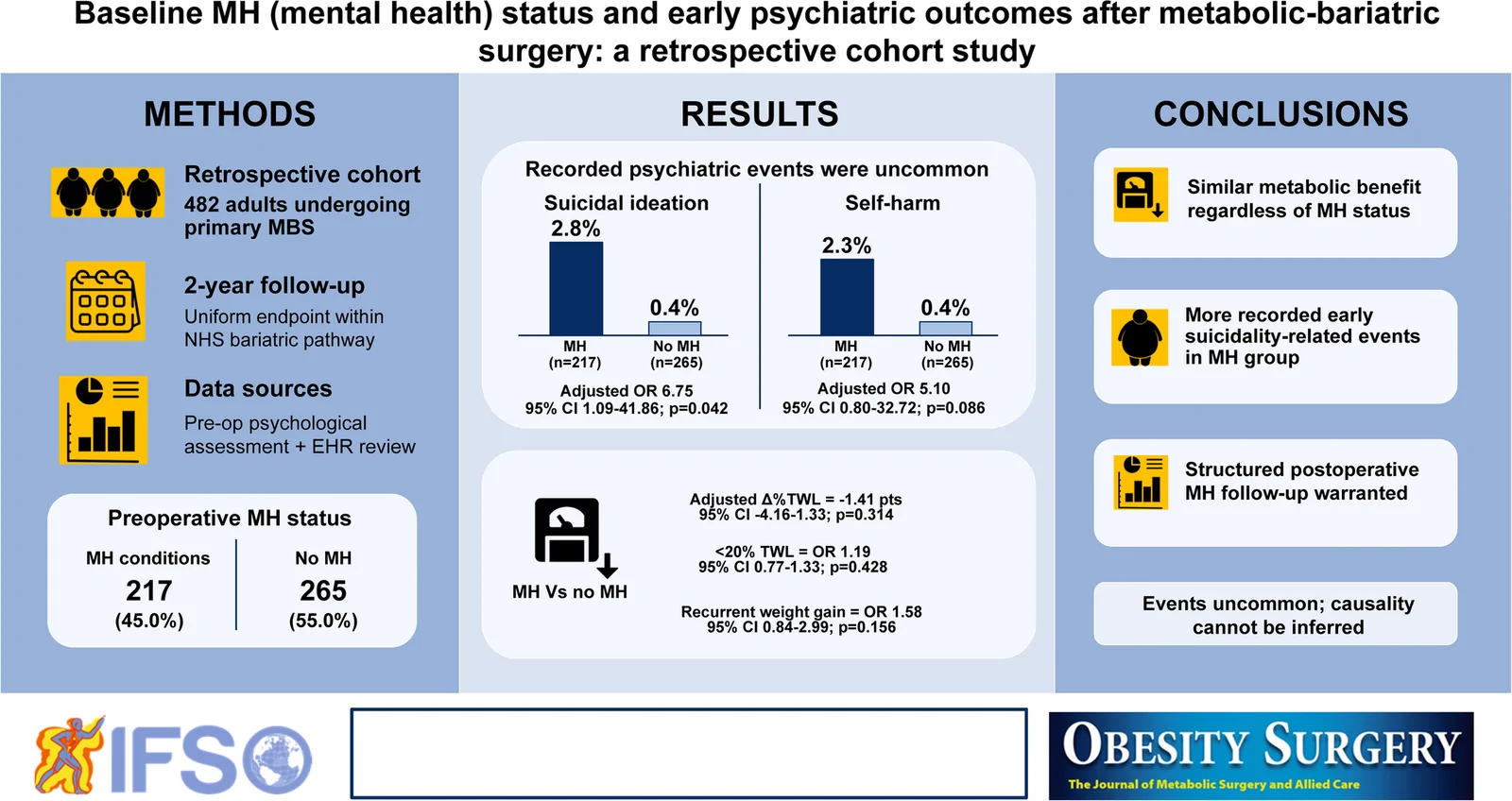

## Highlights

Preoperative mental health (MH) conditions were documented in 45.0% of patients.

At 2 years, suicidal ideation occurred in 2.8% with MH conditions and 0.4% without.

Adjusted odds of suicidal ideation were 6.75x higher in people with MH conditions.

Two-year weight-loss and metabolic outcomes were similar between groups.

## Introduction

Obesity and its metabolic complications represent a major global health burden. Although pharmacological options for obesity have expanded, metabolic–bariatric surgery (MBS) remains the most effective and durable intervention for severe obesity, achieving sustained weight loss and substantial reductions in morbidity and mortality [1]. However, despite these well-established physical health benefits, emerging evidence indicates that the relationship between MBS and mental health (MH) outcomes is more complex and nuanced [2, 3].

Up to half of MBS candidates have co-existing depression, anxiety, eating disorders, or substance-use disorders [3–5]. These conditions can influence postoperative recovery, adherence to follow-up, and quality of life. Evidence suggests that symptoms of depression and anxiety often improve in the first year after surgery [6], yet other data indicate an increased risk of self-harm and suicide during long-term follow-up [7]. The coexistence of metabolic improvement and psychiatric vulnerability illustrates a complex and incompletely understood relationship between physical and MH after MBS [8]. As a result, it remains unclear whether pre-MBS psychiatric morbidity confers additional risks and whether metabolic outcomes influence these associations.

We conducted a retrospective cohort study of adults undergoing MBS to compare psychiatric and metabolic outcomes according to baseline MH status. We aimed to investigate associations between pre-existing MH conditions and post-MBS outcomes to inform targeted postoperative surveillance strategies.

## Methods

### Study Design and Participants

We conducted a retrospective cohort study of adults undergoing primary MBS at a high-volume tertiary MBS unit, between April 2021 and March 2024. The cohort included consecutive patients with 2 years of follow-up, with clinical, demographic, anthropometric and outcome data collected prospectively and recorded in the hospital’s submissions to the National Bariatric Surgery Registry. Preoperative MH conditions were identified from the structured psychological/psychiatric assessment undertaken as part of the routine bariatric pathway and from review of the electronic health records. Postoperative psychiatric outcomes were ascertained retrospectively through review of electronic health records during the 2-year follow-up period. No study-specific face-to-face interviews, telephone assessments, or email questionnaires were conducted.

This study was reported in accordance with STROBE guidelines [9]. Inclusion criteria were patients aged over 18 years, who underwent primary MBS for treatment of obesity [10]. Exclusion criteria included patients who: had primary MBS before 2021; underwent previous bariatric or metabolic procedures (including insertion of an adjustable gastric band); received a cancer diagnosis within 2 years before or after surgery; or underwent surgery for indications other than those listed above. Patients who became pregnant within 2 years post-operatively were excluded. Patients undergoing revisional surgery or lacking baseline data for psychiatric or metabolic outcomes were also excluded.

The primary exposure was the presence of a MH condition prior to MBS, defined as any documented diagnosis of a mood, anxiety, psychotic, personality, or eating disorder identified during preoperative psychiatric assessment. Personality traits were not systematically assessed using validated instruments and were not included in the analysis.

Primary psychiatric outcomes were recorded suicidal ideation, self-harm (including attempted suicide), alcohol misuse, and illicit substance use. In addition, psychotropic medication use was recorded at baseline and at two years after surgery, and discontinuation was assessed among baseline users.

Secondary surgical and metabolic outcomes included percentage total weight loss (%TWL), suboptimal response defined by %TWL < 20%, recurrent weight gain, change in HbA1c, and discontinuation of glucose-lowering, antihypertensive, lipid-lowering, and GLP-1 receptor agonist therapy.

### Procedures

All patients were assessed preoperatively by a multidisciplinary team comprising bariatric surgeons, endocrinologists, psychologists/psychiatrists, specialist nurses, dietitians and anaesthetists. All patients completed a structured preoperative pathway including nutritional optimisation, psychological screening and medical evaluation. All patients underwent a structured preoperative psychological assessment. Preoperative records were also reviewed for documented suicidal ideation and self-harm.

Postoperative care included follow-up for 2 years (which is the duration supported by commissioning within the National Health Service in the UK). Weight-loss and metabolic outcomes were assessed at two years, as this was a uniform postoperative time point with complete data across the cohort. This time point also captured outcomes beyond the initial phase of rapid postoperative weight loss and metabolic improvement and coincided with completion of the routinely commissioned NHS bariatric follow-up pathway. Earlier assessments occurred at variable intervals and were not sufficiently complete for longitudinal analysis. Postoperative care comprised routine follow-up by the bariatric multidisciplinary team, with referral to psychology or psychiatry when clinically indicated. Some patients identified during preoperative assessment as requiring closer MH monitoring had planned psychological or psychiatric review after surgery; however, this was not a standardised pathway applied to all patients. New formal psychiatric diagnoses and the clinical evolution of pre-existing MH conditions were not systematically collected. Psychotropic medication use was recorded at baseline and at two years after surgery.

Surgical procedures included laparoscopic Roux-en-Y gastric bypass (RYGB), laparoscopic sleeve gastrectomy (SG) and laparoscopic one anastomosis gastric bypass (OAGB), which were performed in a single centre by five bariatric surgeons using standardised techniques.

### Statistical Analysis

Continuous variables are presented as mean ± SD and categorical variables as counts and percentages within groups. Standardised mean differences (SMDs) were calculated to assess baseline differences between patients with and without MH conditions, with SMDs < 0.10 considered negligible, 0.10–0.20 small, and > 0.20 indicating increasing imbalance.

Unadjusted differences in %TWL were explored using one-way and two-way analyses of variance (ANOVA). One-way ANOVA compared %TWL by surgery type (SG vs. RYGB+OAGB) in the overall cohort, while two-way ANOVA examined the main effects of surgery type and MH status and their interactions. Homogeneity of variances was assessed using Bartlett’s test, and where heteroskedasticity was present, findings were confirmed using linear regression with heteroskedasticity-robust standard errors.

Fisher’s exact test was used for unadjusted comparisons of proportions between groups. Binary outcomes were analysed using multivariable logistic regression; for rare outcomes, Firth penalised logistic regression was used to reduce small-sample bias and separation. Results are presented as odds ratios (ORs) with 95% confidence intervals (CIs). Continuous outcomes were analysed using multivariable linear regression and are presented as adjusted mean differences with 95% CIs.

All multivariable models were adjusted for age, sex, baseline BMI, and surgery type (SG vs. RYGB+OAGB). Outcome-specific analyses were conducted using available data; no imputation of missing values was performed. A two-sided p-value < 0.05 was considered statistically significant. All analyses were performed using Stata version 19.5 (StataCorp LLC, College Station, TX, USA).

## Results

Of 639 patients assessed for eligibility, 482 met the prespecified criteria and were included in the final cohort (Table 1). Of these, 217/482 (45.0%) had at least one documented preoperative MH condition. Among patients with MH conditions, the most common diagnoses were depressive disorders (35.0%), comorbid anxiety and depression (23.5%), and anxiety disorders alone (12.4%). Severe psychiatric conditions were uncommon, with psychotic or schizophrenia-spectrum disorders present in < 2% and personality disorders in < 1% of patients with MH conditions. Multiple diagnoses were recorded in 20.2% of patients with MH conditions (Table 2). No patient with postoperative suicidal ideation or self-harm had a documented baseline history of these outcomes in the preoperative psychological assessment or available records.

**Table 1:** Baseline characteristics of the study cohort

Variable	*n* (%) or mean ± SD
Age (years)	44.6 ± 11.5
Sex	
Women	393 (81.5%)
Men	89 (18.5%)
BMI (kg/m^2^)	45.1 ± 8.2
Operation type	
SG	264 (54.8%)
RYGB	207 (42.9%)
OAGB	11 (2.3%)
Type 2 diabetes	132 (27.4%)
Hypertension	191 (39.7%)
Dyslipidaemia	121 (25.2%)
HbA1c (mmol/mol)	41.0 ± 11.0
Preoperative previous alcohol misuse	21 (4.4%)
Preoperative previous illicit substance abuse	8 (1.7%)

*BMI* body mass index, *SG* Sleeve gastrectomy, *OAGB* one anastomosis gastric bypass, *RYGB* Roux-en-Y gastric bypass

**Table 2:** Baseline psychiatric diagnoses among patients with ≥ 1 MH condition

Diagnosis type	*n*	%
Anxiety disorder	27	12.4
Depressive disorder	76	35.0
Comorbid anxiety and depression	51	23.5
Binge-eating disorder (treated)	8	3.7
Schizophrenia / schizoaffective disorder	3	1.4
Personality disorder (including borderline type)	1	0.5
Other psychotic disorder	1	0.5
*Other psychiatric diagnosis	6	2.8
**Multiple diagnoses	44	20.2

*Other psychiatric diagnoses include bipolar disorder, obsessive-compulsive disorder, panic disorder and post-traumatic stress disorder

**Multiple diagnoses refer to patients with two or more co-existing psychiatric disorders other than comorbid anxiety and depression

Baseline demographic and metabolic characteristics stratified by MH status are shown in Table 3. Overall, the groups were broadly similar with respect to age, BMI, type 2 diabetes, hypertension, dyslipidaemia, baseline HbA1c, and surgery type (all SMDs < 0.20 except female sex). Mean age was 43.6 ± 11.1 years in the MH group and 45.4 ± 11.7 years in the non-MH group (SMD = − 0.16), while mean BMI was 45.8 ± 8.6 kg/m² and 44.6 ± 7.8 kg/m², respectively (SMD = 0.15). The MH group included a higher proportion of female participants (88.0% vs. 76.2%; SMD = − 0.31). Baseline HbA1c was 40.2 ± 10.0 mmol/mol in the MH group and 41.6 ± 11.7 mmol/mol in the non-MH group (SMD = − 0.13). The proportion undergoing gastric bypass surgery (RYGB or OAGB) was similar between groups (45.2% vs. 45.3%; SMD = − 0.002). Preoperatively, previous history of alcohol misuse was documented in 21/482 patients (4.4%) and illicit substance use in 8/482 patients (1.7%).

**Table 3:** Baseline characteristics among groups

Variable	Patients with ≥ 1 MH condition (*n* = 217)	Patients with no MH condition (*n* = 265)	SMD	*p*-value
Age: mean ± SD (years)	43.6 ± 11.1	45.4 ± 11.7	-0.16	0.0880
BMI: mean ± SD (kg/m²)	45.8 ± 8.6	44.6 ± 7.8	+ 0.15	0.1015
Female sex (%)	88.0	76.2	-0.31	**0.0009**
Type 2 diabetes (%)	25.8	28.8	-0.07	0.5381
Hypertension (%)	35.0	43.6	-0.18	0.0613
Dyslipidaemia (%)	24.4	25.8	-0.03	0.8329
HbA1c mean ± SD (mmol/mol)	40.2 ± 10.0	41.6 ± 11.7	-0.13	0.1574
Gastric bypass surgery (%)	45.2	45.3	-0.002	> 0.9999

*BMI* body mass index, *MH* mental health conditions, *SMD* Standardised Mean Difference. SMD < 0.10 indicates negligible imbalance, 0.10–0.20 indicates mild imbalance, > 0.20–0.30 indicates moderate imbalance

### Psychiatric Outcomes

Postoperative psychiatric events were uncommon overall (Table 4). Suicidal ideation was recorded in 6/217 (2.8%) patients with ≥ 1 preoperative MH condition and 1/265 (0.4%) patients without such conditions (*p* = 0.049). Self-harm events, including suicide attempts, were recorded in 5/217 (2.3%) and 1/265 (0.4%) patients, respectively (*p* = 0.0950). Alcohol misuse was recorded in 5/217 (2.3%) versus 3/265 (1.1%) patients (*p* = 0.4760), and illicit substance use in 4/217 (1.8%) versus 1/265 (0.4%) patients (*p* = 0.1120).

**Table 4:** Adjusted psychiatric outcomes two years after MBS

Outcome	≥ 1 MH condition pre-MBS (*n* = 217)	No MH condition pre-MBS (*n* = 265)	OR (95%CI)	*p*-value
Suicidal ideation	6 (2.8%)	1 (0.4%)	6.75 (1.09–41.86)	**0.0420**
Self-harm (including suicide attempt)	5 (2.3%)	1 (0.4%)	5.10 (0.80–32.72)	0.0860
Alcohol misuse	5 (2.3%)	3 (1.1%)	2.71 (0.66–11.05)	0.1650
Illicit drug use	4 (1.8%)	1 (0.4%)	4.78 (0.68–33.34)	0.1140

Values represent numbers of patients (percentage within group). The model was adjusted for age, sex, baseline BMI, and surgery type

*MBS* metabolic-bariatric surgery, *MH* mental health, *OR* Odds Ratio

In adjusted analyses, the presence of a preoperative MH condition was associated with higher odds of postoperative suicidal ideation (OR 6.75, 95% CI 1.09–41.86; *p* = 0.0420). A similar direction of association was observed for self-harm (including suicide attempt) (OR 5.10, 95% CI 0.80–32.72; *p* = 0.0860), although the estimate was imprecise and did not reach statistical significance. There was no clear evidence of an association between preoperative MH condition and postoperative alcohol misuse (OR 2.71, 95% CI 0.66–11.05) or illicit drug use (OR 4.78, 95% CI 0.68–33.34).

At baseline, 136/482 (28.2%) patients were receiving psychotropic medication, including 98/217 (45.2%) with ≥ 1 preoperative MH condition and 38/265 (14.3%) without. Among the 38 patients receiving psychotropic medication without a documented MH condition, the recorded indications were chronic or neuropathic pain in 23 patients (60.5%), insomnia in 11 (28.9%), and anxiety or depressive symptoms without a formal psychiatric diagnosis in 4 (10.5%). Patients with preoperative MH conditions were less likely to discontinue psychotropic medication during the follow-up period (OR 0.34, 95% CI 0.17–0.68; *p* = 0.0030).

### Weight Loss and Metabolic Outcomes

All 482 patients completed the 2-year clinical follow-up. Complete longitudinal weight data were available for 371 patients (77.0%), while 111 (23.0%) had at least one missing weight measurement during follow-up. Missingness did not differ significantly by preoperative MH status (21.1% in patients without MH conditions vs. 25.4% in those with MH conditions; *p* = 0.274).

Mean %TWL at two years was 24.6 ± 14.3%. Weight-loss outcomes were similar in patients with and without ≥ 1 preoperative MH condition. In adjusted analyses, preoperative MH status was not associated with %TWL at two years (adjusted mean difference − 1.41% points, 95% CI − 4.16 to 1.33; *p* = 0.3140), suboptimal weight-loss response (%TWL < 20%; OR 1.19, 95% CI 0.77–1.87; *p* = 0.4280), or recurrent weight gain (OR 1.58, 95% CI 0.84–2.99; *p* = 0.1560).

Metabolic outcomes at two years were otherwise broadly comparable between groups. Preoperative MH status was not associated with change in HbA1c (adjusted mean difference − 0.22, 95% CI − 3.91 to 3.46; *p* = 0.9080), discontinuation of glucose-lowering medication (OR 0.71, 95% CI 0.46–1.08; *p* = 0.1070), insulin (OR 0.88, 95% CI 0.52–1.49; *p* = 0.6350), GLP-1 receptor agonist therapy (OR 0.98, 95% CI 0.85–1.12; *p* = 0.7640), or antihypertensive medication (OR 0.81, 95% CI 0.51–1.28; *p* = 0.3710). Patients with preoperative MH conditions had lower odds of discontinuing lipid-lowering therapy (OR 0.42, 95% CI 0.21–0.87; *p* = 0.0190).

## Discussion

This single-centre cohort study provides a two-year evaluation of early psychiatric and metabolic outcomes following MBS. Despite equivalent weight and metabolic benefits between patients with and without MH conditions, pre-existing psychiatric illness presented with more recorded suicidality-related events during follow-up.

According to the American Psychiatric Association, individuals pursuing MBS exhibit a substantially higher burden of psychiatric disorders (most notably depression, anxiety, and binge-eating disorder) compared with the general population [11]. After MBS, many patients experience an early improvement in MH symptoms, particularly within the first postoperative year, a period that typically corresponds with the most substantial weight loss [11–13]. At the same time cohort studies and meta-analyses have raised concerns about postoperative psychiatric risk, including substance-use disorders and suicidal or self-harm behaviours, over longer follow-up [2, 3, 8, 14–16]. Our findings are consistent with that broader literature, while adding a more specific clinical observation: with a contemporary bariatric cohort, documented baseline MH conditions may help identify patients who warrant closer postoperative monitoring. The present data do not suggest that poorer weight-loss response explains the psychiatric signal, which is in keeping with previous reports showing that BMI reduction may not account for postoperative mental distress, including in cohorts where patients who later self-harmed achieved similar or greater weight reduction [16, 17]. Previous studies have commonly evaluated changes in psychiatric symptoms or MH service use before and after MBS [12, 13, 15], or compared patients undergoing MBS with matched non-surgical controls [16]. Our study provides a complementary perspective by stratifying 2-year postoperative outcomes according to documented preoperative MH status.

In this cohort, patients with preoperative MH conditions were less likely to discontinue psychotropic medication after surgery. This finding should be interpreted cautiously, as medication continuation may represent appropriate maintenance treatment rather than persistent or worsening psychiatric symptoms. MBS primarily treats obesity and its metabolic complications and does not directly treat underlying psychiatric disorders. Conditions such as depression and anxiety arise through complex biopsychosocial processes and may therefore require continued pharmacological or psychological treatment despite successful postoperative weight loss and metabolic improvement [8]. Postoperative psychological adjustment and other biological and psychosocial changes may also influence MH outcomes [18–21]. These considerations support integrated postoperative follow-up incorporating surgical, endocrine, and MH care, while recognising that the mechanisms underlying psychiatric outcomes were not directly assessed in this study [8, 22].

Current recommendations from the American Society for Metabolic and Bariatric Surgery, the European Association for the Study of Obesity, and the American Gastroenterological Association emphasise alcohol abstinence or careful moderation after MBS [23–25]. In the present cohort, postoperative alcohol misuse was uncommon and no clear association with preoperative MH status was identified. Nevertheless, alcohol-related problems may develop gradually, and adherence to postoperative advice may diminish over time [26]. The two-year follow-up window used in this study may therefore underestimate the longer-term burden of alcohol-related morbidity.

Likewise, findings relating to illicit substance use were imprecise and should be considered exploratory. Although no clear association with preoperative MH status was observed, existing literature suggests that substance-related problems may emerge in a subset of patients following MBS and may reflect pre-existing behavioural vulnerability [27, 28]. Therefore, these findings support careful preoperative assessment and integrated postoperative follow-up for patients with MH or addiction-related needs.

Several limitations should be considered. Given this was an observational cohort, causal inference is limited. Despite the overall cohort size and relatively balanced exposure groups, postoperative psychiatric events were rare. Consequently, the adjusted effect estimates had wide confidence intervals and should be regarded as imprecise and potentially unstable. Firth penalised regression reduced small-sample bias but could not compensate for the limited number of events. Weight loss and metabolic outcomes were evaluated at a single postoperative time point. Consequently, the study could not examine individual trajectories, the timing of metabolic changes, or variability in outcomes during follow-up. Although earlier measurements were available for some patients, their timing and completeness varied, and their inclusion could have introduced bias related to differential follow-up and missing data. MH status encompassed heterogeneous diagnostic categories and did not capture severity or active symptom burden. Notably, no documented baseline suicidality-related history was identified among patients who developed postoperative suicidal ideation or self-harm, although undocumented prior symptoms cannot be excluded. Postoperative psychiatric events were identified through routine clinical documentation rather than structured assessment, and some under-reporting or differential surveillance of patients with known MH conditions is possible. New formal psychiatric diagnoses and changes in pre-existing MH conditions, including improvement, persistence, worsening, or remission, were not systematically assessed. In addition, the intensity of postoperative MH follow-up varied according to clinical need, which may have contributed to differential ascertainment of psychiatric events. Personality traits, which may influence postoperative behaviours and weight-loss trajectories, were not systematically assessed. Residual confounding remains a concern. Although models adjusted for major demographic, metabolic and procedural variables and incorporated rare-event correction, unmeasured factors such as illness severity, psychosocial stressors, informal support systems or access to psychiatric care could still influence the observed associations. In addition, multiple outcomes were analysed without formal correction for multiplicity; therefore, secondary and borderline findings should be considered hypothesis-generating.

In conclusion, in this tertiary bariatric cohort, documented preoperative MH conditions were not associated with poorer postoperative weight-loss or metabolic outcomes, but suicidality-related events were recorded more often in this group during follow-up. These findings support structured postoperative MH surveillance within bariatric multidisciplinary pathways, while also indicating that patients with MH comorbidity should not be assumed to derive less metabolic benefit from surgery. Larger multicentre studies with longer follow-up are needed to confirm and refine these observations.

## Data Availability

No datasets were generated or analysed during the current study.
